# Prevalence of Psychosocial Issues Among Pregnant Women Who Do and Do Not Use Illicit Substances

**DOI:** 10.21203/rs.3.rs-2845911/v1

**Published:** 2023-04-24

**Authors:** Loren S Kock, Heidi S Melbostad, Sarah H Heil

**Affiliations:** University of Vermont

**Keywords:** Pregnancy, illicit drug use, psychosocial issues, population survey

## Abstract

**Objective:**

It is often believed that pregnant women who use illicit substances are more likely to experience psychosocial issues like smoking, depression, and inadequate health care compared to pregnant women who do not. However, the prevalence of these psychosocial issues has rarely been calculated and compared using nationally representative data.

**Methods:**

Important psychosocial issues identified by the American College of Obstetricians and Gynecologists were operationalized using variables in the National Survey on Drug Use and Health. We report weighted prevalence and age-adjusted odds ratios for these issues in pregnant women who did vs. did not report past-month illicit substance use.

**Results:**

Pregnant women (n = 3,657) who reported past-month illicit substance use (6.3%; 95% CI 5.4–7.0) had significantly higher rates of almost all psychosocial issues examined, including past-month cigarette smoking (44.9% vs. 6.5%; age-adjusted odds ratio (AOR) = 7.14 (95% CI 4.98–10.20)); past-month alcohol use (36.1% vs. 7.8%; AOR = 6.80 (4.69, 9.86)); serious past-month distress (23.0% vs. 5.0%; AOR = 4.99 (3.07–8.11)); no health insurance (11.7% vs. 6.2%; AOR = 1.79 (1.07–2.99)); and receipt of food stamps (45.0% vs. 24.0%; AOR = 2.26 (1.55–3.29)). Moving 3 + times in the past year followed a similar pattern, but results were compatible with there being no difference between groups (10.6% vs. 5.5%; AOR = 1.59 (0.95–2.66)). In contrast to other issues examined, English language proficiency was higher among those who reported illicit substance use (4.7% vs. 0.4%; AOR = 0.08 (0.01–0.63)).

**Conclusions:**

Pregnant women who use illicit substances experience higher rates of most psychosocial issues compared to those who do not, reinforcing recommendations for multidisciplinary approaches to care.

## Introduction

The American College of Obstetricians and Gynecologists (ACOG) recommends prenatal care be initiated in the first trimester and continue at regular intervals throughout pregnancy to reduce the risk of adverse outcomes (American College of Pediatrics and American College of Obstetricians and Gynecologists, 2017). Pregnant women who report use of illicit substances initiate prenatal care later than those who do not (second vs. first trimester, respectively) and are more likely to receive inadequate prenatal care (Simmons & Austin, 2022; Wu et al., 2013). One common explanation for these differences is that pregnant women who report use of illicit substances have a higher prevalence of psychosocial issues than pregnant women who do not, which make it more difficult for them to access and engage in care. The extant data on the prevalence of psychosocial issues among pregnant women who report use of illicit substances frequently come from studies on other topics where select issues are reported on when characterizing the population and from studies using convenience samples. Using weighted estimates from a nationally representative population health survey in the United States, this report aims to quantify and compare the prevalence of a range of psychosocial issues faced by pregnant women who did vs. did not report past-month illicit substance use.

## Methods

### Design

Data covered the period 2015 to 2019 and came from the public use files of the National Survey of Drug Use and Health (NSDUH). NSDUH is an annual epidemiological survey of the prevalence of substance use and mental health issues in the US civilian noninstitutionalized population aged 12 or older (SAMHDA, 2019). NSDUH interviews are carried out using primarily computer-assisted personal interviewing and for more sensitive questions related to illicit substance use, using audio computer-assisted self-interviewing. Using a stratified, multi-stage cluster sampling design, approximately 70,000 respondents were interviewed annually (68,073 in 2015, 67,942 in 2016, 68,032 in 2017, 67,791 in 2018, and 67,625 in 2019). Weighted response rates for each year 2015 to 2019 were 69.7%, 68.4%, 67.1%, 66.6% and 64.9%, respectively. Sampling weights were provided by NSDUH to account for unit- and individual-level non-response and adjusted to ensure estimates were consistent with estimates provided by the US Census (SAMHDA, 2019). For the current analysis, respondents were restricted to those who were currently pregnant at the time of the interview (N = 3,657).

### Measures

#### Pregnancy

Women were classified as pregnant if they responded affirmatively to the question “Are you pregnant?”

#### Illicit substance use

We used the recoded variable in the NSDUH survey referring to any reported illicit substance use in the past month (binary: used in past month vs. did not report use in past month) (see Supplementary Table 1). This variable covers past-month use (or misuse in the case of licit/medically prescribed substances) of cocaine, hallucinogens, heroin, inhalants, methamphetamine, marijuana, pain relievers, sedatives, stimulants, and tranquilizers.

#### Psychosocial issues

ACOG recommends that antepartum screening include assessment of multiple psychosocial issues, namely a patient’s desire for pregnancy, tobacco use, substance use, depression, safety, intimate partner violence, stress, barriers to care, unstable housing, communication barriers, and nutrition (American Academy of Pediatrics and American College of Obstetricians and Gynecologists, 2017). We searched the NSDUH codebook for variables that related to these issues. The seven binary (yes/no) variables we identified (Supplementary Table S1) and, in parentheses, the issues they represent are: past-month cigarette smoking (tobacco use), past-month alcohol use (substance use), serious past-month distress (both depression and stress), no health insurance (barriers to care), moved 3 + times in the past year (unstable housing), does not speak English well (communication barriers), and household receives food stamps (nutrition). Past-month distress was measured using the clinically-validated Kessler Psychological Distress Scale (K6) (Kessler et al., 2002, 2010; Prochaska et al., 2012). This six-item measure screens for individuals with a high likelihood of being diagnosed with a mental illness. A sum score with a possible range from 0 to 24 is calculated, with scores of 13 and higher indicating serious distress that warrants intervention. As far as we are aware, there are no variables in NSDUH that relate to a patient’s desire for pregnancy, or safety/intimate partner violence.

#### Analysis

All analyses were carried out in R version 4.2.2, using the *survey* and *tidyverse* packages (Lumley, 2004; Wickham et al., 2019). Weighted sample characteristics are presented for all pregnant women, and for those who did vs. did not report past-month illicit substance use. We then report weighted prevalence statistics with 95% CI for each psychosocial variable among pregnant women who did vs. did not report past-month illicit substance use. To assess the association between each psychosocial issue (binary dependent variable; issue not present (referent) vs. issue present) and past-month illicit substance use (no (referent) vs. yes), separate weighted and age-adjusted logistic regression models for each issue were also run.

Given variation in the legal status of recreational marijuana use between states (Pacula & Smart, 2017), and its relatively higher prevalence compared with other substances in the US (SAMHDA, 2019), we also conducted a sensitivity analysis looking at the prevalence of each psychosocial issue according to use of any illicit substance *other than marijuana* in the past month.

#### Transparency And Openness

All analyses were carried out in R version 4.2.2, using the *survey* and *tidyverse* packages (Lumley, 2004; Wickham et al., 2019) and all data and analysis code are publicly available at the open science framework https://osf.io/394qy/. The data reported in this manuscript were obtained from publicly available data (SAMHSA, https://www.datafiles.samhsa.gov/dataset/national-survey-drug-use-and-health-2020-nsduh-2020-ds0001). A bibliography of journal articles, working papers, conference presentations, and dissertations using NSDUH data is available at https://www.datafiles.samhsa.gov/bibliography-search. The relationships examined and the timeframe over which they are reported, in the present article have not been examined in any previous or current articles, or to the best of our knowledge in any papers that will be under review soon.

## Results

Between 2015–2019, based on an underlying unweighted sample of 3,657 pregnant women in NSDUH, a weighted estimate of 6.7% (95% CI 5.4–7.0) reported using an illicit substance in the past month. Those who reported using at least one illicit substance in the past month were younger, less likely to be Hispanic or college graduates, and more likely to be Non-Hispanic Black/African American, and (Table 1).

The rates of all but one of the psychosocial issues examined were higher among those reporting recent illicit substance use (Table 2). Briefly, among pregnant women who reported using illicit substances in the past month around half reported smoking, one-third reported alcohol use, and one-quarter reported serious psychological distress, respectively. These women had five- to seven-times higher odds of reporting these issues compared with women who did not report using illicit substances (*p* < .001).

In addition, almost half of pregnant women who reported using illicit substances in the past month reported they or someone in their household was receiving food stamps and around one in ten reported that they did not have any health insurance and had moved at least three times in the past year. The odds of these issues were twice as high in the group who did vs. did not report illicit substance use in the past month, although the measure of unstable housing was compatible with their being no association. In contrast, the rate of not speaking English well was higher among those who did not report using illicit substances in the past month. This is likely a reflection of Hispanic women having the highest rate of poor English proficiency (~ 19%) but relatively low rates of past-month illicit substance use (~ 3%) (SAMHDA, 2019).

Results from sensitivity analyses assessing the prevalence of psychosocial issues among those reporting use of any illicit substance *other than marijuana* in the past month were concordant with the primary analyses, albeit with higher point prevalence estimates for some factors such as cigarette smoking, past-month distress, and housing instability (Supplementary Table S2).

## Discussion

In a representative sample of pregnant women in the US between 2015–2019, about 1 of every 15 reported recent use of illicit substances. The prevalence of all but one of the psychosocial issues examined was higher in this group as compared to pregnant women who did not report recent use of illicit substances and typically was at least twice as prevalent, if not more. Although we are not able to quantify how many psychosocial issues each pregnant woman reporting recent use of illicit substances endorsed, the high prevalence of several issues, and the known correlations between them, means it is likely that many women were experiencing more than one and perhaps multiple issues concurrently. The World Health Organization’s most recent guidelines for the identification and management of substance use and substance use disorders (SUDs) in pregnancy say that services for these women “should have a level of comprehensiveness that matches the complexity and multifaceted nature of substance use disorders and their antecedents” (WHO, 2014, p. 7). The present results reinforce long-standing recommendations for multidisciplinary care for this population.

As noted in the Introduction, it is commonly thought that the higher prevalence of many psychosocial issues makes it more difficult for women who use illicit substances to initiate and engage in prenatal care, leading to later and inadequate prenatal care in this population (Simmons & Austin, 2022; Wu et al., 2013). In addition, psychosocial issues may also indirectly contribute to later and inadequate prenatal care. Pregnant women with SUDs often report they feel judged by health care providers (Renbarger et al., 2020) and perinatal providers have reported feeling angry and frustrated with pregnant patients with illicit SUDs and described them as uncaring (Raeside, 2003; Romisher et al., 2018; Seybold et al., 2014). Providers would seem to often be making the fundamental attribution error, attributing women’s actions to their character rather than to external situational factors. As Radcliffe (2011) observed, “few concessions would be seen to be made for the fact that these women...are often contending with the difficult social circumstances that accompany a drug-using or former drug-using lifestyle” (p. 505). Broader appreciation of the difficult circumstances so many of these women are in and compassion for their situation could lead to more women seeking timely and consistent care and in turn, better outcomes.

There are several limitations to the current analysis. First, to maximize the sample for analysis, we computed estimates in those who reported using illicit substances in the past month, rather than only those classified as having an SUD. In addition, most reported substance use refers to marijuana use, which is no longer illicit in many jurisdictions across the US. Nevertheless, results of the sensitivity analysis excluding all reported marijuana use lead to similar, if somewhat less precise, conclusions. Second, accurate measurement of illicit substance use in priority populations in population surveys is challenging due to issues of selective non-response, small case numbers, and under sampling of certain populations such as the unsheltered homeless (Reuter et al., 2021). However, this means the present estimates are likely conservative. Relatedly, two psychosocial issues listed in the ACOG screening guidance that are believed to be more prevalent among women using illicit substances, namely desire for pregnancy and intimate partner violence, do not currently have a related variable in NSDUH. Still, we were able to provide much more precise estimates of many other psychosocial issues by using data from this large population-based survey.

In summary, pregnant women who report using illicit substances experience higher rates of most psychosocial issues compared to those who do not, reinforcing recommendations for multidisciplinary care that matches the complexity of their needs.

## Supplementary Material

Supplement 1

## Figures and Tables

**Table 1:** Characteristics of the sample of pregnant women from the National Survey on Drug Use and Health overall, and by past-month illicit substance use (weighted), 2015–2019

	Overall	Used illicit substance in past month	
	(N = 2,258,599)	No (n = 2,116,932)	Yes (n = 141,667)
**Characteristic**	% (95% CI)	% (95% CI)	% (95% CI)
**Past-month illicit substance use**	6.3 (5.4–7.3)	-	-
**Age (years)**			
12–17	1.7 (1.4, 2.2)	1.6 (1.2, 2.0)	4.6 (2.6, 8.1)
18–25	30.9 (29.5, 32.4)	29.9 (28.5, 31.3)	46.5 (38.0, 55.3)
26–34	51.7 (49.6, 53.7)	52.4 (50.2, 54.5)	40.4 (32.2, 49.2)
35–49	15.7 (14.1, 17.5)	16.2 (14.5, 18.1)	8.4 (4.7, 14.8)
**Race/ethnicity**			
Non-Hispanic White	54.9 (52.7, 57.1)	54.8 (52.4, 57.1)	56.9 (48.2, 65.2)
Non-Hispanic Black/African American	15.6 (14.2, 17.1)	14.9 (13.4, 16.5)	25.1 (18.8, 32.8)
Non-Hispanic Native American/Alaska	0.7 (0.5, 0.9)	0.6 (0.4, 0.9)	1.7 (0.6, 4.2)
Non-Hispanic Native Hawaiian/Pacific Island	0.5 (0.2, 1.0)	0.5 (0.3, 1.1)	0.0
Non-Hispanic Asian	6.1 (4.7, 7.8)	6.3 (4.8, 8.1)	3.3 (1.1, 9.4)
Non-Hispanic more than one race	2.0 (1.6, 2.5)	1.9 (1.5, 2.4)	3.6 (1.7, 7.6)
Hispanic	20.3 (18.2, 22.5)	21.0 (18.9, 23.3)	9.4 (5.9, 14.5)
**Education**			
12–17 years old	1.7 (1.4, 2.2)	1.6 (1.2, 2.0)	4.6 (2.6, 8.1)
Less than high school	11.5 (10.3, 12.9)	11.2 (10.0, 12.6)	16.1 (11.4, 22.2)
High school graduate	22.6 (20.8, 24.6)	22.4 (20.5, 24.4)	26.3 (19.8, 34.1)
Some college/Associate’s degree	29.6 (27.6, 31.7)	29.1 (27.1, 31.3)	36.5 (29.6, 43.0)
College graduate	34.5 (31.7, 37.4)	35.7 (32.7, 38.8)	16.5 (11.0, 24.2)
**Household income**			
Less than $20,000	18.7 (17.4, 20.1)	18.1 (16.8, 19.5)	28.5 (22.5, 25.4)
$20,000-$49,999	31.3 (29.3, 33.3)	31.0 (29.0, 33.1)	34.6 (28.1, 41.7)
$50,000-$74,999	16.3 (14.6, 18.2)	16.4 (14.6, 18.2)	15.6 (10.4, 22.6)
$75,000 or more	33.7 (31.8, 35.7)	34.5 (32.6, 36.6)	21.4 (15.6, 28.5)

Ns are weighted to refer to the population of the US that respondents in the survey represent.

**Table 2:** National Survey on Drug Use and Health survey estimates for prevalence of available psychosocial factors among pregnant women reporting illicit substance use (yes vs. no) in the past month, 2015–2019

Variable	Prevalence (95%CI)	AOR (95% CI)	P
**Past-month cigarette smoking**			
***Illicit substance use***			
No	9.5% (8.4–10.5)	-	-
Yes	44.9% (37.6–52.1)	7.14 (4.98, 10.20)	< 0.001
**Past-month alcohol use**			
Illicit substance use			
No	7.9% (6.7–9.1)	-	-
Yes	36.1% (29.0–43.2)	6.80 (4.69, 9.86)	< 0.001
**Household receives food stamps**			
Illicit substance use			
No	24.0% (22.3–25.6)	-	-
Yes	45.0% (36.6–53.4)	2.26 (1.55, 3.29)	< 0.002
**No health insurance**			
Illicit substance use			
No	6.2% (5.2–7.1)	-	-
Yes	11.7% (6.6–16.7)	1.79 (1.07, 2.99)	0.027
**Serious past-month distress**			
Illicit substance use			
No	5.0% (4.2–5.7)	-	-
Yes	23.0% (16.3–29.7)	4.99 (3.07, 8.11)	< 0.001
**Moved 3 + times in past year**			
Illicit substance use			
No	5.5% (4.4–6.5)	-	-
Yes	10.6% (6.4–14.8)	1.59 (0.95, 2.66)	0.077
**Does not speak English well**			
Illicit substance use			
No	4.7% (3.7–5.8)	-	-
Yes	0.4% (0.0–1.0)	0.08 (0.01, 0.63)	0.018

Based on an unweighted sample of currently pregnant women = 3,657.

Event rates are based on Ns weighted to be representative of US population (2015–2019).

Adjusted odds ratios (AORs) based on weighted sample, adjusted for age.

## Public Health Significance

This study shows that pregnant women who use illegal drugs are dealing with more issues like cigarette smoking, mental health problems, and unhealthy diets than pregnant women who do not, suggesting they will need more help to have healthy pregnancies.
