# Supplementary material for: Prevalence of Psychosocial Issues Among Pregnant Women Who Do and Do Not Use Illicit Substances
Source: Res Sq. 2023 Apr 24:rs.3.rs-2845911. Preprint. [Version 1] doi: 10.21203/rs.3.rs-2845911/v1 (PMC10168477; doi:10.21203/rs.3.rs-2845911/v1)
Supplement: Supplement 1 [file NIHPPrs2845911v1-supplement-1.pdf]

Supplementary Files

This is a list of supplementary files associated with this preprint. Click to download.

- Supplementary0.2.docx
